# *In Vivo* Anti-Influenza Virus Activity of Japanese Herbal (Kampo) Medicine, “Shahakusan,” and Its Possible Mode of Action

**DOI:** 10.1155/2012/794970

**Published:** 2012-12-26

**Authors:** Rei Hokari, Takayuki Nagai, Haruki Yamada

**Affiliations:** ^1^Graduate School of Infection Control Sciences, Kitasato University, 5-9-1 Shirokane, Minato-ku, Tokyo 108-8641, Japan; ^2^Department of Drug Discovery Sciences, Kitasato Institute for Life Sciences, Kitasato University, 5-9-1 Shirakane, Minato-ku, Tokyo 108-8641, Japan; ^3^Department of Basic Research, Oriental Medicine Research Center, Kitasato University, 5-9-1 Shirokane, Minato-ku, Tokyo 108-8642, Japan

## Abstract

*Background*. A Kampo medicine, Shahakusan (SHS), has been prescribed in late phase of infection that causes inflammations in the lung. But effect of SHS on viral infection in respiratory tract has never been reported. *Objectives*. To evaluate anti-influenza virus activity of SHS and its mode of actions through immune systems. *Methods*. SHS (0.3 g/kg/day) was orally administered to BALB/c miceforupper (URI) or lower respiratory tract infection (LRI) of influenza virus A/PR/8/34. The virus titer of nasal lavage fluid (NLF) at 5 or 2 day postinfection (p.i.) and cytokine mRNA expressions in mandibular lymph node or lung at 5 or 4 day p.i. were evaluated for URI or LRI, respectively. The histopathological examinations of lung tissue and NK cell activity in the splenocytes were also evaluated at 4 day p.i. on LRI. *Results*. When SHS was administered from 7 days before to 4 days p.i. for URI, the virus titer was significantly decreased in comparison with water-treated control, and IL-4, IL-1**β**, and IL-10 mRNA expression was decreased, but IL-12A mRNA expression was increased. Administration of SHS from one day before to one day p.i. for LRI significantly decreased the virus titer. SHS also decreased infiltration of inflammatory cells in the bronchoalveolar spaces and damage of desquamated mucosal epithelia of bronchiole, decreased IP-10 mRNA expression, and increased NK cell activity. *Conclusion*. SHS has no direct effect on influenza virus infection but exerts antiviral effect in mice by its immunomodulating activity through action of NK cells and anti-inflammatory activity in the lung.

## 1. Introduction

Influenza is a viral infection that affects mainly upper respiratory tract. Influenza infection usually lasts for about one to two weeks and is characterized by sudden onset of high fever, followed by myalgia, headache, and severe malaise, nonproductive cough, sore throat, and rhinitis [1]. Most infected people recover within one to two weeks without requiring medical treatment. However, the high risk of complications occurs among children younger than age two, adults older than age 65, and people of any age with underlying diseases such as chronic heart, lung (bronchial asthma, etc.), kidney, liver, blood, and metabolic diseases (diabetes, etc.), or compromised immune systems (AIDS, etc.) [1]. It has been reported that around 80% of mortality by influenza virus infection is caused by pneumonia [2, 3].

Anti-influenza virus drugs can be classified into M2 protein inhibitors such as amantadine and rimantadine, which are effective for only influenza A virus, and inhibitors for influenza virus neuraminidase such as oseltamivir and zanamivir, which have been used for the treatment of influenza A and B viruses. However, occurrence of drug-resistant strains and several side effects associated with these drugs have been reported [3, 4].

Traditional herbal medicines in Far Eastern countries have played an important role in health care of this area, especially in Japan, China, and Korea [5]. Among Japanese herbal (Kampo) medicines, Maoto (Ma-huang-tang in Chinese) is one of the formulas which has traditionally been prescribed to patients with influenza in Japan, and it has reported that Maoto shows an antipyretic effect clinically [6–8]. However, based on traditional Kampo medicine theory, Maoto containing Ephedrae Herba as one of component herbs is not suitable to use for patients with sweating or general debility and is suitable to use for them in early phase of infection.

A Kampo medicine, Shahakusan (SHS, Xie-bai-san in Chinese, Sabaeksan in Korean) was originally described in a classical Chinese book for treatments of infantile diseases, Xiao'er Yaozheng Zhijue (Key to Differentiation and Therapeutics of Children's Diseases) [9] and has been also used in Japan [10] although it is not so popular. SHS has been prescribed for the treatments of infantile pneumonia, bronchitis, and early stage of measles. SHS is composed of four herbs: Mori Cortex, Lycii Cortex, Glycyrrhizae Radix, and Oryzae Fructus, and has been used in late phase of infection that reveals inflammations due to heat accumulating in the lung [9]. The four component herbs show mild actions, thus are considered suitable for patients with general debility such as children and elderly people. Although *in vivo* anti-inflammatory effect of SHS on the lipopolysaccharide (LPS) induced murine acute lung injury model [11], and a few *in vitro* anti-inflammatory activity have been reported [12, 13], effect of SHS on viral infection in respiratory tract has never been reported.

In present study, because SHS had no direct *in vitro* anti-influenza virus effect, we examined *in vivo* its oral antiviral effect in mice. If SHS shows *in vivo* anti-influenza virus activity, it has possibilities to show host-mediated effects such as enhancements of humoral immunity and/or cytotoxic NK cell activity, and/or suppression of inflammation caused by the viral infection. In order to evaluate these hypotheses, the effects of SHS on the immune functions were also studied.

## 2. Materials and Methods

### 2.1. Preparation of SHS Extract and Its Herbal Components

Four medicinal plants were used for preparation of SHS: Mori Cortex (root bark of *Morus alba* Linné, lot no. 0410240, cultivated at Anhui Province in China in 2006), Lycii Cortex (root bark of *Lycium chinense *Miller, lot no. C05321, cultivated at Sichuan Province in China in 2006), Glycyrrhizae Radix (root of *Glycyrriza uralensis *Fisher, lot no. 9B31621, cultivated at inner Mongolia and Ningxia Hui autonomous region in China in 2007–2010), and Oryzae Fructus (caryopsis of *Oryza sativa* Linné, lot no. 039009003, cultivated at Niigata Prefecture in Japan in 2006). Mori Cortex and Glycyrrhizae Radix were purchased from Uchida Wakanyaku Ltd. (Tokyo, Japan), Lycii Cortex was purchased from Tsumura & Co. (Tokyo, Japan), and Oryzae Fructus was purchased from Tochimoto Tenkaido Co. Ltd. (Osaka, Japan). Voucher specimens of these plants were deposited at Laboratory of Biochemical Pharmacology for Phytomedicines, Kitasato Institute for Life Sciences, Kitasato University in Tokyo, Japan. SHS extract was prepared as follows: a mixture of Mori Cortex (4.0 g), Lycii Cortex (4.0 g), Glycyrrhizae Radix (2.0 g), and Oryzae Fructus (2.0 g) was extracted with 600 mL of distilled water until the volume to half. The aqueous extract was filtered (GFP; Kiriyama glass Co., Tokyo, Japan) and the filtrate was lyophilized (yield: 14.4%). The lyophilized material was suspended in distilled water and used for oral administration to mice (0.3 g/kg/day).

### 2.2. Three-Dimensional High Performance Liquid Chromatography (3D-HPLC) Analysis

**Figure 1:**
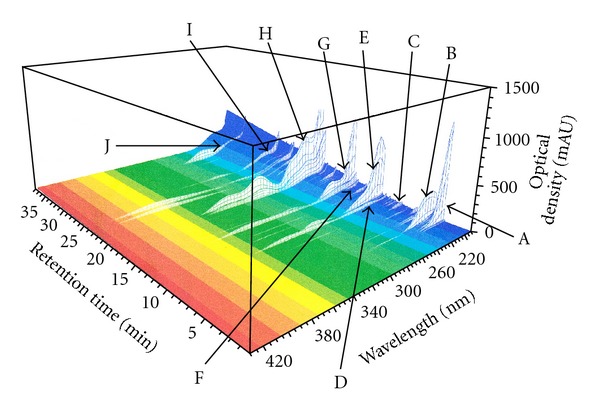
Three-dimensional (3D) HPLC profile of SHS extract. (A), (C), and (F) are unknown peaks derived from Mori Cortex; (B), (D), (E), and (G) are unknown peaks derived from Lycii Cortex; (H) liquiritin (derived from Glycyrrhizae Radix); (I) liquritigenin (derived from Glycyrrhizae Radix); (J) glycyrrhizin (derived from Glycyrrhizae Radix).

SHS extract (10 mg) was dissolved in water (1 mL) with Vortex mixier and sonication. The solution was filtered using a DISMIC-13HP PTFE membrane filter (0.45 *μ*m, Advantec, Tokyo, Japan). Analysis of the filtrate was performed with an Agilent 1100 3D-HPLC system (Agilent Technologies, Inc., Santa Clara, CA, USA) equipped with a photodiode-array detector. A TSK gel ODS-80Ts column (4.6 × 250 mm) (Tosoh Co., Tokyo, Japan) was kept at 40°C. Elution of the mobile phase was carried out by a linear gradient of 10 mM phosphoric acid-acetonitrile (95 : 5 → 5 : 95) for 60 min at a flow rate of 0.8 mL/min. A 3D-HPLC profile of an aqueous SHS extract was shown in Figure 1. The analysis based on ultraviolet (UV) absorption clearly showed the presence of the following major constituents in SHS: liquiritin, liquiritigenin, and glycyrrhizin (originating from Glycyrrhizae Radix), and other constituents were identified their origins of the component herbs by the comparison with 3D-HPLC profile of each aqueous extract from four component herbs. The 3D-HPLC profile clearly showed the major constituents derived from three component herbs except Oryzae Fructus (Figure 1).

### 2.3. Cell, Virus, and Vaccine

Madin-Darby canine kidney (MDCK) cells were grown in Eagle's minimum essential medium (EMEM) (Gibco Invitrogen Co., Carlsbad, CA, USA) containing sodium bicarbonate (2.2 mg/mL), HEPES (0.3%), heat-inactivated fetal bovine serum (10%), penicillin G (100 units/mL), streptomycin (100 *μ*g/mL), and amphotericin B (Fungizone) (2.5 *μ*g/mL) at 37°C with 5% CO_2_. Mouse-adapted influenza virus A/PR/8/34 (H1N1) was maintained in Kitasato Institute for Life Sciences, Kitasato University (Tokyo, Japan). The virus was grown in allantoic cavity of 10-days-old embryonated egg for 48 hours at 34°C. The allantoic fluid was harvested and centrifuged at 1000 ×g for 20 minutes, and then the resulting supernatant was stored in small portions at −80°C. Influenza hemagglutinin (HA) vaccine was prepared from mouse-adapted influenza virus A/PR/8/34 by the method of Davenport et al. [14]. Biotinylation of the HA vaccine was conducted using a biotinylation kit (Sulfo-OSu) (Dojindo Laboratories, Kumamoto, Japan) according to the manufacturer's instructions.

### 2.4. Animals

Specific pathogen-free female BALB/c mice (6-7 weeks old) were purchased from CLEA Japan (Tokyo, Japan). The animals were housed in plastic cages in an air-conditioned room at 23 ± 2°C with a relative humidity of 55 ± 10% under a 12 hours light/dark cycle, fed a standard laboratory diet, and given water ad libitum. Animal experiments were approved by the Animal Research Committee of Kitasato University, and performed in accordance with the Guidelines for Care and Use of Laboratory Animals at Kitasato University and Guidelines for Proper Conduct of Animal Experiments from Science Council Japan.

### 2.5. *In Vitro* Anti-Influenza Virus Experiments

Virus titers of culture supernatant of MDCK cells were estimated by activity of viral neuraminidase in the supernatant [15]. Mouse-adapted influenza virus A/PR/8/34 (H1N1) was used in the *in vitro* antiviral assay. A solution of the SHS extract was prepared in the sterilized water. MDCK cells (3 × 10^5^ cells/mL, 100 *μ*L/well) were placed in a 96-well tissue culture plate (BD Falcon, Franklin Lakes, NJ, USA), and cultured for 2 days at 37°C with 5% CO_2_. The influenza virus was added to confluent monolayers of the cell culture at a multiplicity of infection (MOI) of 0.0001 plaque forming units (PFU)/cell (ca. 11 PFU) in 180 *μ*L of MEM medium containing 2% bovine serum albumin (BSA) and acetyltrypsin (Sigma-Aldrich Co., St. Louis, MO, USA) (3 *μ*g/mL). The samples (20 *μ*L/well, final concentration of 0.39 to 100 *μ*g/mL) or only the sterilized water (control) was added to MDCK cells, and cultured for 3 days at 37°C. An aqueous solution of 0.1 mM 2′-(4-methylumbelliferyl)-*α*-D-*N*-acetylneuraminic acid (4-MU-NeuAc, Toronto Research Chemicals Inc., Toronto, Canada) (50 *μ*L) and phosphate buffered saline (PBS; 50 *μ*L) were added to the culture supernatant (50 *μ*L), and incubated at 37°C for 10 min in Labsystems Fluoroskan II (Dainippon Sumitomo Pharma, Osaka, Japan). The monolayers of MDCK cells in the culture plate were washed with PBS, and the viable cells were determined by a colorimetric method, which is based on the *in situ* reduction of 3-(4,5-dimethylthiazol-2-yl)-2,5-diphenyltetrazolium bromide (MTT) by viable cells [15] according to the procedure of Watanabe et al. [16]. One hundred *μ*L of MTT (1 mg/mL) in PBS was added to each well of the monolayers, and the contents were incubated for 2 hours at 37°C. The resulting formazan precipitate was dissolved with isopropanol containing 0.04 M HCl (100 *μ*L/well), and the absorbance of the solution was determined spectrophotometrically at 570 nm with Infinite 200 M microplate reader (Tecan, Männedorf, Switzerland). Oseltamivir (Tamiflu, Roche, Basel, Switzerland) (20 *μ*L/well, final concentration of 10 *μ*g/mL) was used as a positive control of an antiviral agent for influenza virus A/PR/8/34. For cytotoxicity assay, test sample solutions prepared in the sterilized water, were added to the confluent MDCK cells without virus as described above. The cells were cultured at 37°C with 5% CO_2_ for 3 days, and the number of viable cells was determined using the MTT assay as described above.

### 2.6. *In Vivo* Anti-Influenza Virus Experiments

**Figure 2:**
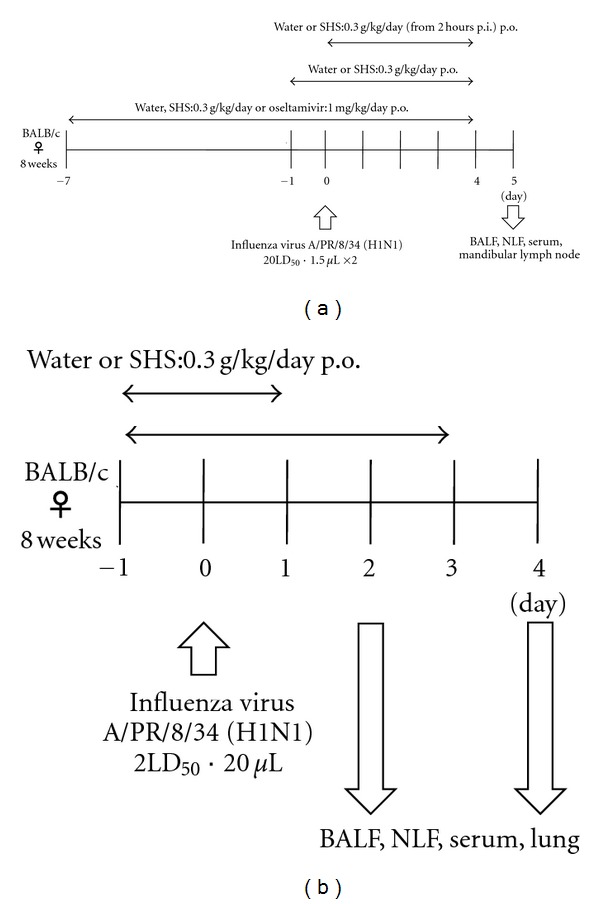
Experiment protocols of drug administrations and influenza virus infection to the upper (a) or lower (b) respiratory tract of mice.

Upper respiratory tract infection of influenza virus [17]: mice were anesthetized by intraperitoneal injection of Somnopentyl (sodium pentobarbital; Merck & Co. Inc., Whitehouse Station, NJ, USA), and then infected intranasally by dropping 1.5 *μ*L of influenza virus suspension (10 LD_50_) in PBS containing 0.1% BSA into each nostril. SHS extract was administered orally to the mouse from 7 days before to 4 days post infection (p.i.), one day before to 4 days p.i. or 2 hours p.i. to 4 days p.i. of the virus (Figure 2(a)). At 5 day p.i., serum specimens were prepared from mice by drawing blood from the infra-axillary artery. A nasal lavage fluid (NLF) was obtained by washing the nasal cavity of the head with 2 mL of PBS containing 0.1% BSA. A bronchoalveolar lavage fluid (BALF) was obtained by injecting 2 mL of the washing solution twice into the trachea and lungs, which were separated from the body. A mandibular lymph node was harvested and stored at −80°C.

Lower respiratory tract infection of influenza virus: mice were anesthetized by intraperitoneal injection of Somnopentyl, and then infected intranasally by dropping 20 *μ*L of influenza virus suspension (2 LD_50_) in PBS containing 0.1% BSA into one nostril. SHS extract was administered orally to the mouse from one day before to one day p.i. or 3 days p.i. of the virus (Figure 2(b)). At 2 day p.i. and 4 day p.i., BALF, NLF, and serum samples were prepared as previously described. The lung specimens were stored at −80°C.

### 2.7. Plaque Assay

Influenza virus titers were determined with plaque assay according to the method described by Kiyohara et al. [18]. Briefly, duplicate cultures of MDCK cells in a 6-well plastic plate (BD Falcon, Franklin Lakes, NJ, USA) were exposed to 100 *μ*L of BALF and NLF samples for 30 minutes at 37°C. The cells were overlaid with 2 mL of medium containing 3 *μ*g/mL acetyltrypsin, 0.01% DEAE-dextran hydrochloride (Sigma-Aldrich Co.), 0.1% glucose, and 0.8% agar (Ina Food Industry Co., Nagano, Japan) followed by cultivation at 37°C for 2 days. The cells were overlaid with 1 mL of the above medium containing 0.01% neutral red (Wako Pure Chemical Industries, Osaka, Japan) and cultured at 37°C for one day. The number of plaques was counted.

### 2.8. Determination of Anti-Influenza Virus Antibodies

The titers of IgA, IgG_1_ and IgM antibodies specific for influenza virus were determined using a reverse-phase fluorescence ELISA [19] as follows. Each well of 96-well Immulon 4 EIA plates (Thermo Fisher Scientific, Waltham, MA, USA) was coated with 100 *μ*L of capture antibody (BD Biosciences Pharmingen, San Jose, CA, USA) (1 *μ*g/mL purified anti-mouse IgA, IgG_1_, or IgM antibody in 0.01 M sodium carbonate-bicarbonate buffer [pH 9.5] containing BSA 10 *μ*g/mL) and incubated at 37°C for 3 hours. Then, the capture antibody was discarded and 300 *μ*L of blocking reagent (1% fat-free milk in PBS) was added to each well and incubated for one hour at 37°C. After incubation, all the wells were washed four times with 200 *μ*L of PBST (PBS with 0.05% Tween 20), and 100 *μ*L of twofold serial dilutions of NLF, BALF, or serum in SuperBlock blocking buffer (Pierce Biotechnology, Rockford, IL, USA) (10-fold diluted with PBS) containing 0.05% Tween 20 was added to each well. The plate was incubated at room temperature overnight and then washed with PBST. Biotinylated HA vaccine (1 *μ*g/mL) in blocking reagent (100 *μ*L) was added to each well and the plate was mixed on a microplate mixer MPX-96 (AGC Techno Glass, Chiba, Japan) at room temperature for one hour. After washing with PBST, 100 *μ*L of streptavidin-*β*-galactosidase (1 : 1000 in blocking reagent; Calbiochem, EMD Chemicals Inc., Darmstadt, Germany) was added to each well. The plate was mixed on a Microplate mixer at room temperature for one hour and washed with PBST, before addition of 100 *μ*L of 0.1 mM 4-MU-*β*-galactoside (Sigma-Aldrich Co.) to each well and incubation at 37°C for 2 hours. Finally, 100 *μ*L of 0.1 M glycine-NaOH (pH 10.3) was added to each well. Fluorometric quantification of 4-methylumbelliferone was carried out at an excitation wavelength of 355 nm and an emission wavelength of 460 nm using Infinite 200 M microplate reader.

### 2.9. NK Cell Activity

The NK cell activity of mouse splenocytes was assessed using flow cytometry [20] as described previously [21]. The level of target cell lysis was determined using a Cytomics FC500 flow cytometer (Beckman Coulter, Brea, CA, USA), and the NK cell activity was expressed as the percentage of effector cell-specific lysis.

### 2.10. Lung Histology

Lung was inflated with 10% formaldehyde solution (Kokusan Chemical Co. Ltd., Tokyo, Japan) and then fixed in the same solution. Preparation of lung tissue paraffin sections stained with hematoxylin and eosin were ordered to Mitsubishi Chemical Medience Co. (Tokyo, Japan).

### 2.11. Quantitation of mRNA Expression in Mandibular Lymph Node and Lung

Total RNA was extracted using Sepasol-RNA I Super G (nacalai tesque, Kyoto, Japan) from the mandibular lymph node and lung. Total RNA (0.5 or 2.0 *μ*g) was then reverse transcribed using ReverTra Ace (TOYOBO, Osaka, Japan) according to the manufacturer's protocol. The resulting cDNA was amplified by the polymerase chain reaction (PCR) technique using SYBR Green Realtime PCR Master Mix Plus (TOYOBO) for quantitative PCR with specific primers according to the manufacture's instructions and determined using a Rotor-Gene Q (QIAGEN, Hilden, Germany). Sequences of the primers are shown in Table 1.

### 2.12. IP-10 (IFN-*γ* Inducible Protein-10, CXCL10) Protein Level in BALF

The protein level of IP-10 in the BALF was determined by using the ELISA kit (R&D Systems Inc., Minneapolis, MN, USA) according to the manufacturer's instructions.

### 2.13. Statistical Analysis

The significance of differences between three experimental groups was analyzed with one-way ANOVA followed by post hoc multiple comparison. The software used was KaleidaGraph Ver.4.0 (Synergy Software, HULINKS Inc., Tokyo, Japan). The significance of differences between two experimental groups was analyzed with an independent *t*-test. A probability (*P*) value *<*0.05 was considered significant.

## 3. Results

### 3.1. Effects of SHS on Influenza Virus Infection in MDCK Cells

**Figure 3:**
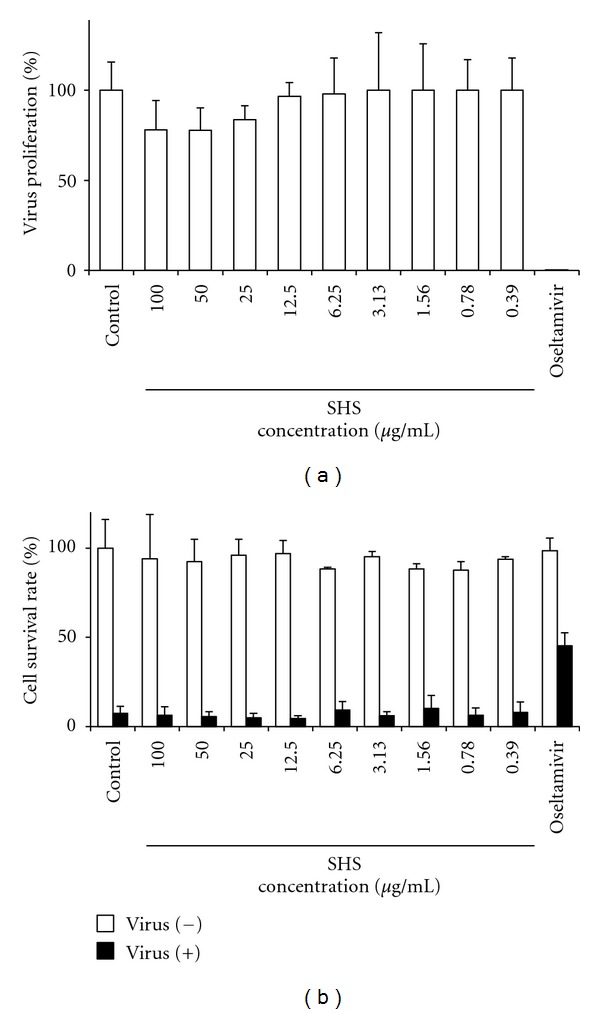
*In vitro* effects of SHS extract on the proliferation of influenza virus A/PR/8/34 (a) and the survival rate of MDCK cells infected or mock-infected influenza virus A/PR/8/34 (b). Values represent means ± S.D. (*n* = 4).

To test the effect of SHS on the proliferation of influenza virus and its cytotoxic activity, the MDCK cells were infected with A/PR/8/34 (H1N1) virus or mock-infected and cultured in the presence of various concentrations of SHS (0.39 to 100 *μ*g/mL) or under a drug free condition. Oseltamivir showed a potent antiviral activity against the viral infection at 10 *μ*g/mL. However, the negligible effects of SHS were shown on the proliferation of virus (Figure 3(a)) and survival of infected MDCK cells (Figure 3(b)), and SHS showed very low cytotoxicity against mock-infected MDCK cells (Figure 3(b)).

### 3.2. Effects of SHS on Upper Respiratory Tract Influenza Virus Infection in Mice

#### 3.2.1. Effect of SHS on the Proliferation of Influenza Virus in the Nasal Cavity of Mice

**Figure 4:**
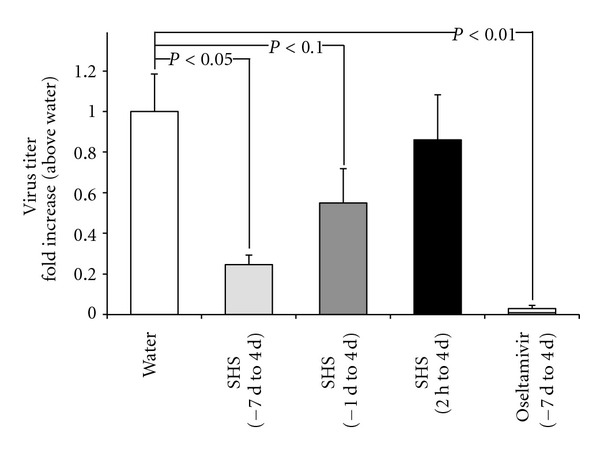
Effect of SHS on the proliferation of influenza virus in the nasal cavity of upper respiratory tract infected mice. BALB/c mice were administered orally with water, SHS or oseltamivir from 7 days before to 4 days p.i. (−7 d to 4 d), one day before to 4 days p.i. (−1 d to 4 d), or 2 hours p.i. to 4 days p.i. (2 h to 4 d) as shown in Figure 2(a). The infectious virus titers in NLF of influenza virus infected mice were estimated at 5 day p.i. with plaque assay. Values represent means ± S.E. (*n* = 9).

When SHS was administered orally to BALB/c mice at a dose of 0.3 g/kg/day from 7 days before to 4 days post infection (p.i.) of influenza virus, the infectious virus titer of the NLF at 5 day p.i. was decreased significantly compared to the water-treated group (Figure 4). Oseltamivir showed a potent antiviral effect under the same condition. When SHS was administered to the mice at the same dose from one day before to 4 days p.i., the virus titer of the NLF observed a tendency of decrease compared with the water-treated control. However, when SHS was administered orally to the mice at the same dose from 2 hours p.i. to 4 days p.i., no antiviral effect was observed (Figure 4).

#### 3.2.2. Effects of SHS on Anti-Influenza Virus Antibody Titers of Mice

At 5 day p.i., the anti-influenza virus IgA antibody titers in NLF and BALF, IgG_1_ antibody titers in serum and BALF, and IgM antibody titers in NLF were not enhanced on SHS-treated mice in comparison with those of water-treated group (data not shown).

#### 3.2.3. Effects of SHS on Cytokine mRNA Expressions in the Mandibular Lymph Nodes of Mice

**Figure 5:**
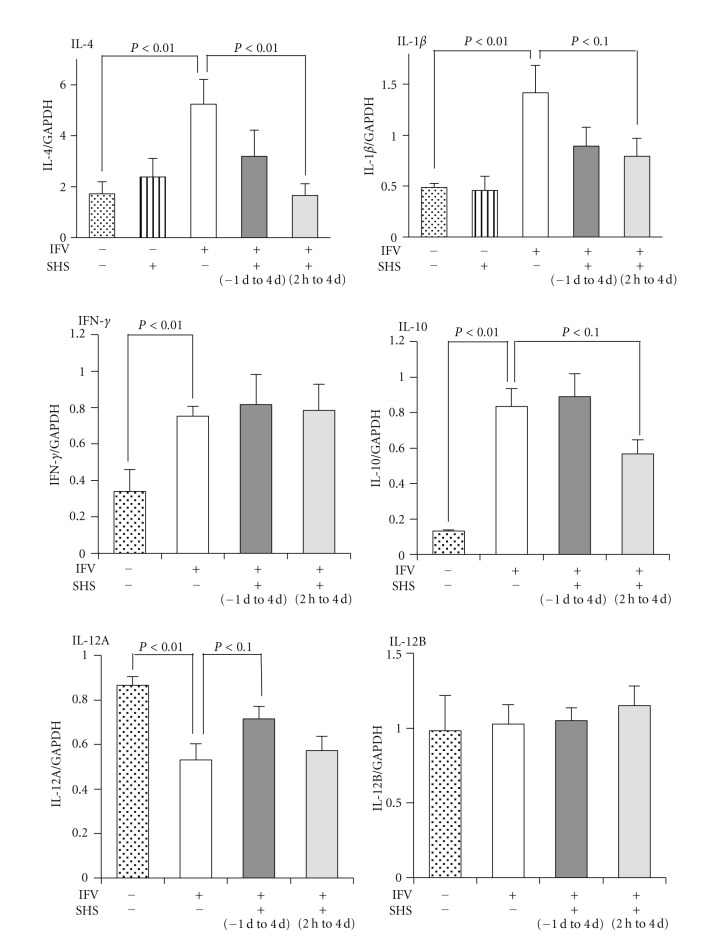
Effects of SHS on IL-4, IL-1*β*, IFN-*γ*, IL-10, IL-12A, and IL-12B mRNA expressions in the mandibular lymph nodes of influenza virus infected mice (upper respiratory tract infection). BALB/c mice were administered orally with water or SHS from one day before to 4 days p.i. (−1 d to 4 d), or 2 hours p.i. to 4 days p.i. (2 h to 4 d), as shown in Figure 2(a). The mRNA expressions in the mandibular lymph nodes of influenza virus infected mice were estimated at 5 day p.i. and those of mock-infected mice were estimated at next day after 5 days administration (same duration from 2 hours p.i. to 4 days p.i.) of water or SHS with real-time PCR. Values represent means ± S.E. (*n* = 9).

To understand the responses to SHS treatment on cytokine mRNA level, real-time quantitative PCR analyses of the mandibular lymph nodes were performed. At 5 day p.i., IL-4, IL-1*β*, IFN-*γ*, and IL-10 mRNA expressions on influenza virus-infected mice were significantly increased in comparison with those of mock-infected groups, but the IL-12A mRNA expression was decreased significantly (Figure 5).

When SHS was administered orally from 2 hours p.i. to 4 days p.i., IL-4 mRNA expression was decreased significantly in comparison with that of water-treated group. IL-1*β* mRNA expression showed a tendency of decrease by the administration of SHS. Whereas oral administration of SHS for 5 days (same duration from 2 hours p.i. to 4 days p.i.) did not affect IL-4 and IL-1*β* mRNA expressions in mock-infected mice (Figure 5). IL-10 mRNA expressions also showed a tendency of decrease by the administration of SHS from 2 hours p.i. to 4 days p.i. On the contrary, IL-12A mRNA expression showed a tendency of increase when SHS was administrated from one day before to 4 days p.i. compared with the water-treated group (Figure 5). However, IFN-*γ* and IL-12B mRNA expressions on SHS-treated mice were not changed in comparison with those of water-treated group.

### 3.3. Effects of SHS on Lower Respiratory Tract Influenza Virus Infection in Mice

#### 3.3.1. Effects of SHS on the Proliferation of Influenza Virus in the Nasal and Bronchoalveolar Cavities of Mice

**Figure 6:**
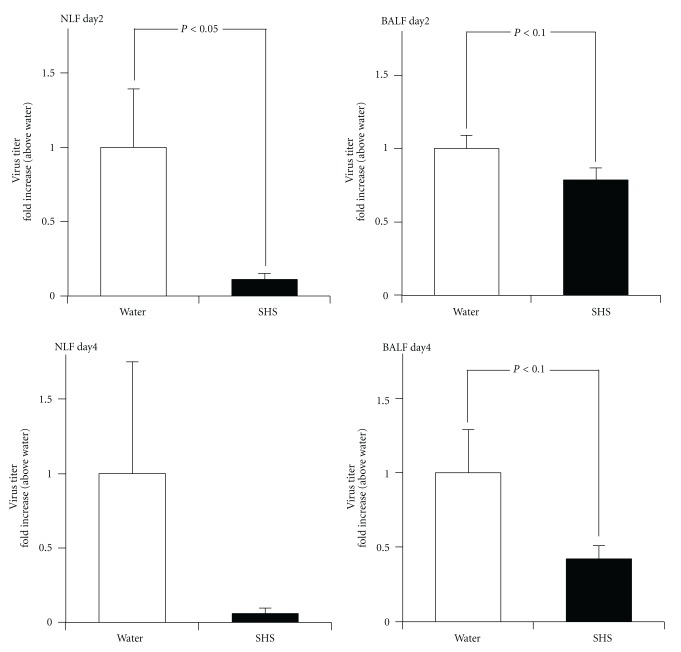
Effects of SHS on the proliferation of influenza virus in the nasal and bronchoalveolar cavities of lower respiratory tract infected mice. BALB/c mice were administered orally with water or SHS from one day before to one day p.i. or 3 days p.i. as shown in Figure 2(b). The infectious virus titers in NLF and BALF of influenza virus infected mice were estimated at 2 day p.i. or 4 day p.i. with plaque assay. Values represent means ± S.E. (*n* = 18).

When SHS was administered orally to the mice at a dose of 0.3 g/kg/day from one day before to one day p.i., the infectious virus titer of the NLF was significantly reduced than that of water-treated group at 2 day p.i. (Figure 6). When SHS was administered orally to the mice at the same dose from one day before to one day p.i. or 3 days p.i. (Figure 2(b)), the virus titer of the BALF showed a tendency of decrease compared to those of water-treated group both at 2 day p.i. and 4 day p.i. (Figure 6). However, the virus titer of NLF from SHS-treated mice at 4 day p.i was seemed to decrease in comparison with that of water-treated group, but it was not significant.

#### 3.3.2. Effects of SHS on Anti-Influenza Virus Antibody Titers of Mice

At 4 day p.i., SHS did not enhance the anti-influenza virus IgA antibody titers in NLF and BALF, IgG_1_ antibody titers in serum and BALF, and IgM antibody titers in BALF of the virus infected mice compared to water as control (data not shown).

#### 3.3.3. Effect of SHS Treatment on the Lung Tissue of Mice

**Figure 7:**
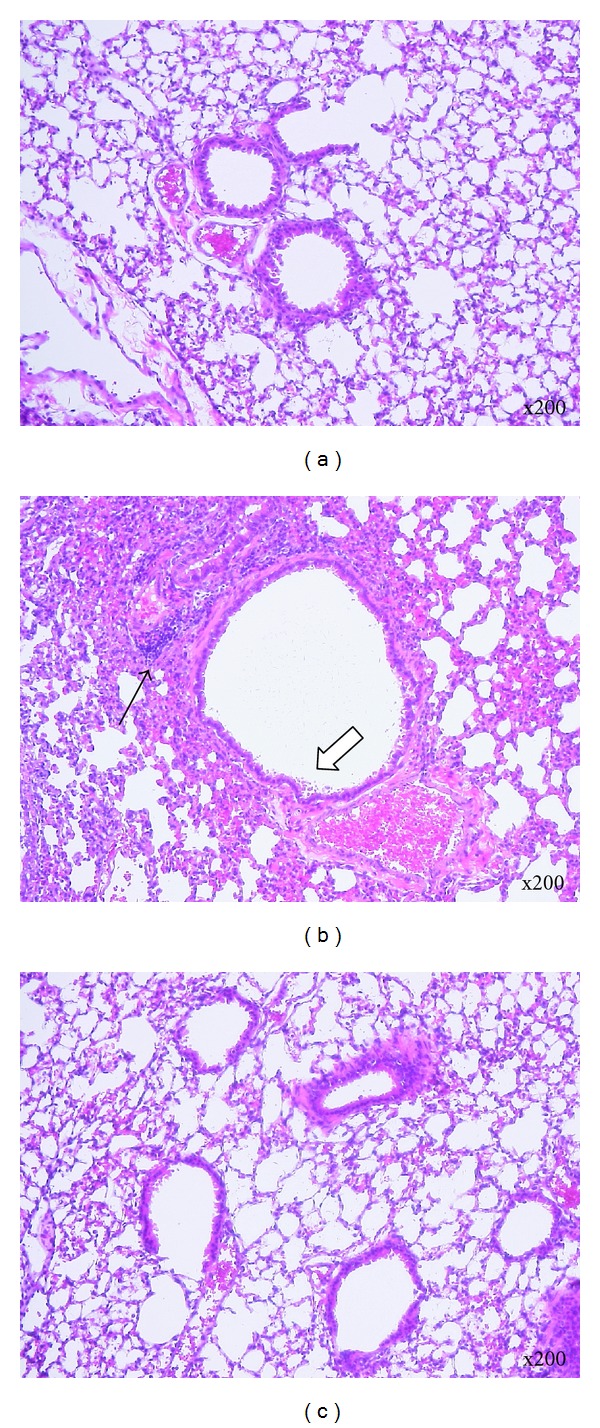
Effect of SHS treatment on the lung tissue of influenza virus infected mice (lower respiratory tract infection). Representative histologic sections of lungs harvested at 4 day p.i. and stained with hematoxylin and eosin. Mock-infected BALB/c mice were administered orally with water as a control (a), and influenza virus infected mice were administered orally with water (b) or SHS (c) from one day before to 3 days p.i. as shown in Figure 2(b). Thin arrow indicates infiltration of inflammatory cells. Open arrow indicates desquamated mucosal epithelia. Magnification 200x.

At 4 day p.i., representative histological sections of lungs were harvested and stained with hematoxylin and eosin. The histological image of the control lung exhibited normal pulmonary and bronchiolar architectures and resident cells (Figure 7(a)). Influenza virus infection induced marked infiltration of inflammatory cells on the perivascular and peribronchiolar areas (Figure 7(b), thin arrow), bronchial dilatation, and desquamated mucosal epithelia of bronchioles in the lung tissue (Figure 7(b), open arrow). However, these influenza virus induced pathological findings in lung were attenuated by the administration of SHS (Figure 7(c)) as similar as these of control mock-infected mice.

#### 3.3.4. Effects of SHS on IP-10 (IFN-*γ* Inducible Protein-10, CXCL10) mRNA Expression in the Lung and IP-10 Protein Level in BALF of Mice

**Figure 8:**
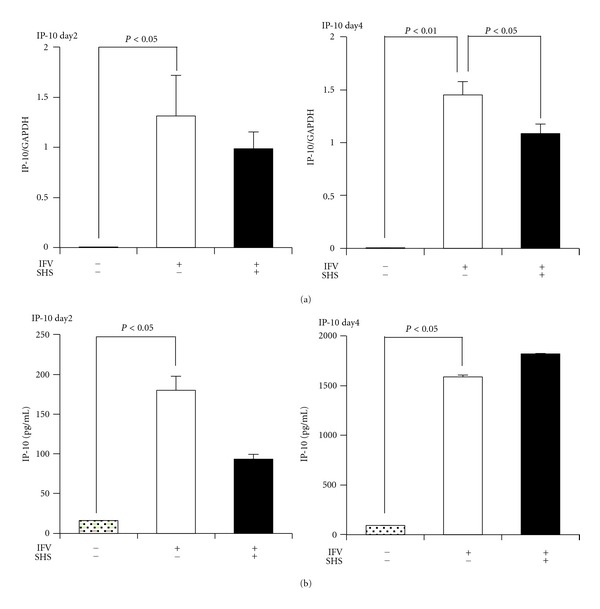
Effects of SHS on IP-10 mRNA expression in the lung (a) and IP-10 protein level in BALF (b) of influenza virus infected mice (lower respiratory tract infection). BALB/c mice were administered orally with water or SHS from one day before to one day p.i. or 3 days p.i. as shown in Figure 2(b). The IP-10 mRNA expression in the lung of influenza virus infected mice was estimated at 2 day p.i. and 4 day p.i. with real-time PCR. The protein level of IP-10 in BALF of influenza virus infected mice was estimated at 2 day p.i. and 4 day p.i. with ELISA. Values represent means ± S.E. (*n* = 6).

Influenza virus infection significantly enhanced IP-10 mRNA expression in the lungs and IP-10 protein level in BALF of mice both at 2 day p.i. and 4 day p.i. compared with those of mock-infected mice (Figure 8). However, IP-10 mRNA expressions in the lung were seemed to decrease by the administration of SHS compared to the water-treatment at 2 day p.i. and 4 day p.i., but significant decrease was shown only at 4 day p.i. (Figure 8(a)). IP-10 protein level in BALF was also seemed to decrease by the administration of SHS compared to the water-treatment only at 2 day p.i., but the decrease was not significant (Figure 8(b)).

#### 3.3.5. Effects of SHS on the NK Cell Activity in Splenocytes of Mice

**Figure 9:**
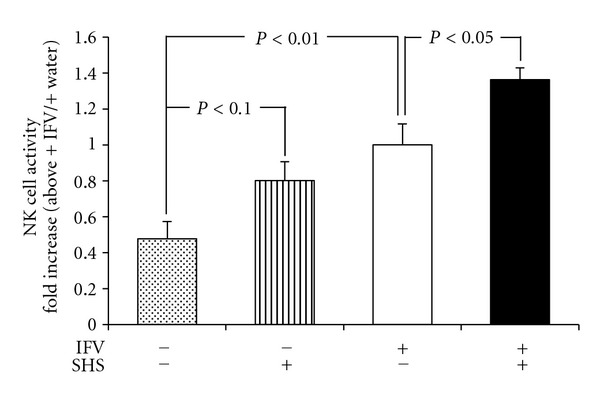
Effects of SHS on the NK cell activity in splenocytes of influenza virus infected (lower respiratory tract infection) or non-infected mice. BALB/c mice were administered orally with water or SHS for 5 days, or water or SHS from one day before to 3 days p.i. (5 days) as shown in Figure 2(b). The NK cell activities in splenocytes were determined at 4 day p.i. with flow cytometry. Values represent means ± S.E. (*n* = 5).

When SHS was administered orally for 5 days without influenza virus infection, a tendency of increase of NK cell activity in splenocytes was observed in comparison with that of the water-administered control (Figure 9). When influenza virus was infected to the mice, the NK cell activity in SHS-treated mice was enhanced significantly compared to that of water-treated group at 4 day p.i. (Figure 9).

## 4. Discussion

Some of Kampo medicines have been reported their *in vivo* effects for influenza. Antipyretic potency of Maoto (Ma-huang-tang in Chinese) in human [8, 22] and Gingyosan (Yin-qiao-san in Chinese) in mice [23, 24] have been reported, Kakkonto (Ge-gen-tang in Chinese) has increased IL-12 production in the BALF of mice [25], and Shoseiryuto (Xiao-qing-long-tang in Chinese) has augmented anti-influenza virus IgA antibody followed by alleviation of the proliferation of influenza virus in the respiratory tract of mice [26–28]. Based on Kampo medicine theory, these formulas have been used in early phase of infection. It has also been reported that Hochuekkito (Bu-zhong-yi-qi-tang in Chinese), which has been used for the recovery of debilitating condition, has anti-influenza virus activity in mice [29], and Unpito (Wen-pi-tang in Chinese) for the recovery of fatigue condition alleviates inflammation in the lung of influenza virus infected mice by inhibition of xanthine oxidase (XOD) [30].

Shahakusan (SHS) has been used in late phase of infection that caused inflammations due to heat accumulating in the lung [9], and this formula shows mild actions, thus is considered suitable for patients with general debility such as children and elderly people. However, effect of SHS for influenza virus infection has never been reported.

Present study clarified that SHS had a negligible effect on the proliferation of virus and survival of infected MDCK cells (Figure 3), suggesting that SHS has no direct effect for influenza virus infection. However, present study clearly showed that the prior oral administration of SHS can protect the proliferation of influenza virus A/PR/8/34 (H1N1) in the respiratory tract of mice (Figures 4 and 6), but SHS did not enhance antiviral antibodies in NLF, BALF and serum of the infected mice both on the upper and lower respiratory tract infections. These results suggest that SHS shows host-mediated anti-influenza virus activity through the action mechanism which is different from that of Shoseiryuto.

Present study also showed that IL-1*β* mRNA expression in mandibular lymph node (one of regional lymph node of nasopharynx) was increased by influenza virus infection but downregulated by the administration of SHS from 2 hours p.i. to 4 days p.i. on upper respiratory tract infection (Figure 5). A proinflammatory cytokine IL-1*β* is an important mediator of the inflammatory response, and is involved in a variety of cellular activities, including cell proliferation, differentiation, and apoptosis [31]. IL-1*β* is also readily produced by influenza A virus-infected leukocytes [32]. The report for anti-inflammatory effect of SHS on the LPS induced murine lung injury model also showed decrease of IL-1*β* mRNA expression in lung and protein level in BALF [11]. In present study, SHS alone did not affect the IL-1*β* mRNA expression in mandibular lymph node of mock-infected mice (Figure 5). These results suggest that decrease of IL-1*β* mRNA expression by the action of SHS in present study may occur as a result of suppression of inflammation in nasal cavity but not direct suppression by SHS. With these results, SHS may have the potential to suppress inflammation due to influenza virus infection. IL-4 is a cytokine that induces differentiation of naive helper T cells (Th0 cells) to Th2 cells [33]. IL-10 is produced primarily by monocytes and a lesser extent by lymphocytes, and downregulates the expression of Th1 cytokines [34]. It has been reported that when the influenza virus was infected to the mice, increases of IL-4 and IL-10 mRNA expressions were observed in the lung [35]. In present study, IL-4 and IL-10 mRNA expressions also increased in the mandibular lymph node of mice by influenza virus infection, but SHS did not affect the IL-4 mRNA expression in mock-infected mice (Figure 5). IL-12 is a heterodimeric cytokine encoded by two separate genes, IL-12A (p35) and IL-12B (p40). It is known that IL-12 activates on T and NK cells, and has broad biological activities. This cytokine has been found to be important for sustaining a sufficient number of memory/effector Th1 cells to mediate long-term protection to an intracellular pathogen [36] and induces a cell-mediated immune response [37]. Present study showed that IL-4 and IL-10 mRNA expressions were downregulated, and IL-12A mRNA expression was upregulated in mandibular lymph node by SHS administration to the influenza virus-infected mice (Figure 5). These results suggest that SHS measurably influenced Th1/Th2 balance and activated Th1 cells in nasopharynx. SHS may inhibit the proliferation of influenza virus in nasal cavity by the activation of cell-mediated immune response through the activation of Th1 cells. In present study, administration of SHS from one day before the virus infection inhibited the proliferation of influenza virus in nasal cavity at 5 day p.i., but the administration from 2 hours p.i. did not inhibit on upper respiratory tract infection (Figure 4). IL-12A mRNA expression was increased when SHS was administered from one day before to 4 days p.i. on upper respiratory tract infection (Figure 5). Therefore, these results suggest that the increased IL-12A mRNA expression involves in the antiviral effect of SHS as a possible action. It has been reported that when another Kampo medicine, Kakkonto was administered orally to the mice from one day before influenza virus infection, the level of IL-12 in the BALF was increased on 2 day p.i. and virus yield in BALF was reduced on 3 day p.i. [25]. These observations suggest that at least a part of the anti-influenza virus activities of SHS and Kakkonto cause by upregulation of IL-12 on respiratory tract.Further, bronchial asthmatic patients are one of groups at high risk of influenza virus infection, and IL-4 is a typical Th2-type cytokine that plays a key role in human allergic asthmatic responses [38]. The decrease of IL-4 mRNA expression by the action of SHS suggests that SHS also has a potential to protect and/or treat diseases such as severe asthma.

The histological experiment suggests that SHS has an anti-inflammatory effect in the lung of mouse on the lower respiratory tract infection (Figure 7). IP-10 (CXCL10) is a Th1 cell-related chemokine which is important in the recruitment of Th1 cells involved in host immune defense against intracellular pathogens such as viral infection [39]. IP-10 expression level is known to associate with inflammatory diseases including infectious diseases [40]. The decrease of this chemokine mRNA expression was observed in the lung of SHS-treated mice on lower respiratory infection (Figure 8). Therefore these results suggest that SHS shows anti-inflammatory effect through the decrease of IP-10 in the lung.

Present study also showed that oral administration of SHS augmented NK cell activity in splenocyte of the lower respiratory tract infected mice and also non-infected mice (Figure 9). In this respect, SHS might augment cellular immunity and protect influenza virus infection, because NK cells are lymphocytes of the innate immune system that play a crucial role in the early host defense against various virus infections [41, 42]. We could not measure NK cell activity in the lung of influenza virus-infected mice after SHS administration in present study, because it was hard to collect enough amounts of lymphocytes from lung of mouse for NK cell assay. Elucidation of effect of SHS on NK cell activity in the lung must await further study.

Glycyrrhizin is a major component of Glycyrrhizae Radix, one of component herbs of SHS, and its *in vivo *anti-influenza virus activity has been reported as a result of i.p. administration to the mice [43]. However, *in vivo* anti-influenza virus activities of the other component herbs of SHS have never been reported. Although oral effect of glycyrrhizin has not been reported, it has a possibility to be one of active substances in SHS. Elucidation of orally active anti-influenza substances in SHS and further action mechanism of anti-influenza virus activity of SHS are now in progress by our group.

## 5. Conclusions

Consequently, this study shows that SHS has *in vivo* anti-influenza virus activity for the first time. In addition, SHS exerts its anti-influenza virus effects in mice through its immunomodulating activity such as NK cell activity in splenocytes and anti-inflammatory activity in lung.

## Figures and Tables

**Table 1:** Primers sequences used for real-time PCR.

Gene	Primer		Length
GAPDH	Forward	TGTCCGTCGTGGATCTGAC	75
	Reverse	CCTGCTTCACCACCTTCTTG	
IFN-*γ*	Forward	TACTGCCACGGCACAGTCATTGAAA	144
	Reverse	GCTCTGCAGGATTTTCATGTCACCA	
IL-1*β*	Forward	TGTTCTTTGAAGTTGACGGACCCCA	108
	Reverse	TGATGTGCTGCTGCGAGATTTGA	
IL-4	Forward	CCAGCTAGTTGTCATCCTGCTCTTC	150
	Reverse	ACGTTTGGCACATCCATCTCCG	
IL-10	Forward	TTTGAATTCCCTGGGTGAGAA	98
	Reverse	GCTCCACTGCCTTGCTCTTATT	
IL-12A	Forward	CCATCAGCAGATCATTCTAGACAA	146
	Reverse	GGATGCAGAGCTTCATTTTCA	
IL-12B	Forward	TGAACTGGCGTTGGAAGCACGG	102
	Reverse	TGCAAGTTCTTGGGCGGGTCTG	
IP-10	Forward	TGAGGGCCATAGGGAAGCTTGAAAT	109
	Reverse	TCCGGATTCAGACATCTCTGCTCAT	
